# Visual Body Part Representation in the Lateral Occipitotemporal Cortex in Children/Adolescents and Adults

**DOI:** 10.1093/texcom/tgaa007

**Published:** 2020-04-13

**Authors:** Yuko Okamoto, Ryo Kitada, Takanori Kochiyama, Hiroaki Naruse, Kai Makita, Motohide Miyahara, Hidehiko Okazawa, Hirotaka Kosaka

**Affiliations:** 1 Advanced Telecommunications Research Institute International, Seika-cho, Sorakugun, Kyoto 619-0288, Japan; 2 Brain Activity Imaging Center, ATR-Promotions, Seika-cho, Sorakugun, Kyoto 619-0288, Japan; 3 School of Social Sciences, Nanyang Technological University, 48 Nanyang Avenue, HSS-04-13, Singapore 639818; 4 Division of Physical Therapy and Rehabilitation, University of Fukui Hospital, Eiheiji-cho, Yoshida-gun, Fukui 910-1193, Japan; 5 Research Center for Child Mental Development, University of Fukui, Eiheiji-cho, Yoshida-gun, Fukui 910-1193, Japan; 6 Department of Clinical Psychological Science, School of Medicine, Hirosaki University, 1 Bunkyo-cho, Hirosaki, Aomori, 036-8560, Japan; 7 Biomedical Imaging Research Center, University of Fukui, Eiheiji-cho, Yoshida-gun, Fukui, 910-1193 Japan; 8 Department of Neuropsychiatry, Faculty of Medical Sciences, University of Fukui, Eiheiji-cho, Yoshida-gun, Fukui 910-1193, Japan

**Keywords:** development, functional magnetic resonance imaging, representational similarity analysis, sensory characteristics

## Abstract

The lateral occipitotemporal cortex (LOTC) that responds to human bodies and body parts has been implicated in social development and neurodevelopmental disorders like autism spectrum disorder (ASD). Neuroimaging studies using a representational similarity analysis (RSA) revealed that body representation in the LOTC of typically developing (TD) adults is categorized into 3 clusters: action effector body parts, noneffector body parts, and face parts. However, its organization of younger people (i.e., children and adolescents) and its association with individual traits remain unclear. In this functional MRI study, TD adults and children/adolescents observed photographs of hands, feet, arms, legs, chests, waists, upper/lower faces, the whole body, and chairs. The univariate analysis showed that fewer child/adolescent participants showed left LOTC activation in response to whole-body images (relative to those of chairs) than adult participants. Contrastingly, the RSA on both age groups revealed a comparable body representation with 3 clusters of body parts in the bilateral LOTC. Hence, this result indicates that, although response to whole-body images can differ, LOTC body part representations for children/ adolescents and adults are highly similar. Furthermore, sensory atypicality is associated with spatial LOTC organization, suggesting the importance of this region for understanding individual difference, which is frequently observed in ASD.

## Introduction

The face and other body parts convey social information such as identity, emotion, and intention. Face and body part recognition is a basic social skill that emerges at infancy and matures until adulthood (Weigelt et al. 2014; Bank et al. 2015; Hock et al. 2017). For instance, along with other categories of objects, visual discrimination performance for the face and body improved from age 5 to age 10 (Weigelt et al. 2014). Despite its relevance, the neural mechanisms underlying developmental changes of body recognition are not fully understood.

Previous functional magnetic resonance imaging (fMRI) studies identified the extrastriate body area (EBA) in the lateral occipitotemporal cortex (LOTC), which responds to the perception of nonface body parts compared to other objects such as outdoor scenes or tools (Downing et al. 2001, 2006). Several fMRI studies examined if EBA activation differed between children, adolescents, and adults (Peelen et al. 2009; Pelphrey et al. 2009; Ross et al. 2014, 2019). Peelen et al. (2009) conducted an fMRI study where 7- to 17-year-old and adult participants watched static pictures. Both groups showed stronger responses to body parts than to tools in the LOTC, whereas the size of the right body-sensitive activation (EBA) was reduced during development. Another study in which the participants observed static pictures showed that 7- to 11-year-old children showed adult-like activation in the LOTC (Pelphrey et al. 2009). In the study by Ross et al. (2014), 6- to 11-year-old children and adults passively watched short videos of body or object movements. The results showed that the EBA was larger in adults than in children (Ross et al. 2014). In their later study, the authors further examined developmental EBA changes by measuring the neural response to movies of emotional or natural body movements in 6- to 11-year-old children, 12- to 17-year-old adolescents, and adults. They found that body-sensitive activation increased throughout development, whereas children and adolescents showed adult-like emotional modulation of the EBA (Ross et al. 2019). These studies consistently report that school-age children showed body-sensitive activation in the LOTC, although the developmental change in EBA size is still controversial.

These developmental studies mainly focused on body-sensitive responses by comparing the observed body parts with objects in other categories. More specifically, body-sensitive regions were depicted by comparing a whole body with nonbody objects (Ross et al. 2014, 2019) or by contrasting nonface body parts with faces (Peelen et al. 2009; Pelphrey et al. 2009). However, another line of studies on typically developing (TD) adults demonstrated that spatial patterns of LOTC activation differ even among body parts (Orlov et al. 2010; Bracci et al. 2015). For instance, Bracci et al. (2015) conducted a representational similarity analysis (RSA) to examine the organization of body part representations in the LOTC of TD adults. The representation structures in the LOTC were related to functional-semantic properties of the body parts organized into 3 clusters: (1) body parts used as action effectors (hands, feet, arms, and legs), (2) noneffector body parts (chests and waists), and (3) face parts (upper and lower faces) (Bracci et al. 2015). This body representation organization in the LOTC appears reasonable because this region is involved in action observation and execution (Astafiev et al. 2004; Gazzola and Keysers 2009; Oosterhof et al. 2012), gestural interaction (Okamoto et al. 2014; Sasaki et al. 2018), and understanding the meaning of action (Kubiak and Króliczak 2016; Wurm et al. 2016, 2017). Given the findings in previous developmental studies, we expect that body parts are represented in the LOTC similarly between children/adolescents and adults. However, to our knowledge, no previous study has tested body part representation organization in the LOTC of TD children and adolescents.

The present study utilized fMRI to examine whether body part representation in the LOTC of TD children/adolescents (9–15 years old) is also organized into the 3 aforementioned body part clusters. The participants observed photographs displaying a hand, foot, arm, leg, chest, waist, upper face (UF), lower face (LF), the whole body, or a chair. After revealing LOTC activation using pictures of a person’s whole body (relative to that of a chair), an RSA and a classification-based multivariate pattern analysis were conducted to examine body part representation in the LOTC.

The present study further focused on individual differences in body part representation in the LOTC, as our previous studies revealed reduced LOTC activation in individuals with autism spectrum disorder (ASD) (Okamoto et al. 2014, 2017, 2018). For instance, when participants observed pictures of nonface body parts, a difference in the EBA activation was detected between children with and without ASD, but not between adults with and without ASD (Okamoto et al. 2014). Most previous research focused on social-communicative difficulties or other cognitive functions such as imagination or attention as a feature of ASD. However, hyper- or hypo-reactivity to sensory input or unusual interests in sensory aspects were added to the diagnostic criteria when the Diagnostic and Statistical Manual of Mental Disorders was revised in 2013 (DSM-5, APA 2013). Although motor difficulties are not included in the diagnostic criteria, 79% of ASD individuals meet the developmental coordination disorder (DCD) criteria (Green et al. 2009). Thus, it is reasonable to assume that sensory-motor dysfunction is a feature of ASD. According to the concept of heterogeneities (Ecker and Murphy 2014; Lombardo et al. 2019), some ASD individuals have more severe difficulties in social communication than those have sensory-motor difficulties, whereas other ASD individuals exhibit the opposite pattern. Notably, the LOTC is activated by visual stimuli (Downing et al. 2001), motor execution (Astafiev et al. 2004), and social interaction (Sasaki et al. 2018). Thus, it is unclear which feature is associated with LOTC function. Even in the TD population, some people have a higher indication of autistic traits than others (Lombardo et al. 2019), thus it is possible to examine association of neural underpinning and individual difference observed ASD among TD individual. Therefore, an exploratory analysis was also conducted to examine characteristics associated with body part representations in the LOTC of TD child and adolescent participants.

## Material and Methods 

### Participants

In the present study, 27 young adults and 24 children/adolescents were recruited. One participant of the adult group and 2 participants of child/adolescent group were excluded from further analyses based on the exclusion criteria (i.e., a history of major medical or neurological illness including epilepsy, significant head trauma, or a lifetime history of alcohol and drug dependence). Therefore, the data of 26 adults (age, mean ± standard deviation [range]: 23.8 ± 3.4 [20–31] years) and 22 children/adolescents (age, 11.8 ± 1.9 [9–15] years) were analyzed (Table 1). General autistic traits, sensory and motor characteristics, and intellectual abilities were measured in the child/adolescent group. General autistic traits were determined using the social responsibleness scale (SRS; Kamio et al. 2013; Constantino and Gruber 2005) and the autism spectrum quotient (AQ; Auyeung et al. 2008). Sensory-motor atypicality is a characteristic of individuals with ASD; however, these functions are not included in the AQ or SRS. Therefore, sensory characteristics were measured using the sensory profile (SP), which is a relevant tool to understand a child’s sensory processing patterns in everyday situations (Dunn 1999; Ito et al. 2013). Notably, SP and SP-based questionnaires are frequently used to evaluate ASD sensory characteristics of (Kientz and Dunn 1997; Watling et al. 2001; Nieto et al. 2017; Avery et al. 2018; Schulz and Stevenson 2019). Motor abilities were evaluated using the Developmental Coordination Disorder Questionnaire (DCDQ; Wilson et al. 2007; Nakai et al. 2011) and the Movement Assessment Battery for Children, Second Edition (MABC-2; Henderson 2007); both of these tools are used to evaluate DCD motor difficulties, including ASD motor abilities (Miyachi et al. 2014; Hirata et al. 2015). Intellectual ability was assessed as the full-scale intelligent quotient (FSIQ) using the Wechsler Intelligence Scale for Children, fourth edition (Wechsler 2004; Table 1). The present protocol was approved by the Ethics Committees of the University of Fukui (Japan) and the Advanced Telecommunications Research Institute International (Japan). The study was conducted in accordance with the Declaration of Helsinki. After the study had been explained in detail, written informed consent was obtained from each participant or in the case of children and adolescents, from their legal guardians.

**Table 1:** Demographic data

	Adult group	Child/adolescent group
Age (years)	23.8 ± 3.4	11.8 ± 1.9
Sex (male/female)	14/12	10/12
Handedness (right/left)	24/2	19/3
FSIQ		99.3 ± 11.2
SRS score		23.6 ± 11.3
AQ total score		10.2 ± 5.2
SP		
Low registration score		18.4 ± 3.8
Sensory seeking score		30.1 ± 7.2
Sensory sensitivity score		24.0 ± 4.1
Sensation avoiding score		40.0 ± 7.9
MABC-2 total score (standard score)		11.9 ± 2.3
DCDQ total score		61.1 ± 10.9

Notes: Handedness was assessed using the Edinburgh Handedness Inventory (Oldfield 1971). FSIQ: full-scale intelligence quotient of the Wechsler Intelligence Scale for Children—fourth edition (WISC-IV; Wechsler 2004), SRS: social responsibleness scale (Constantino and Gruber 2005; Kamio et al. 2013), AQ: autism spectrum quotient (Auyeung et al. 2008), SP: sensory profile (Dunn 1999; Ito et al. 2013), MABC-2: movement assessment battery for children—second edition (Henderson 2007), DCDQ: developmental coordination disorder questionnaire (Wilson et al. 2007; Nakai et al. 2011). Each score is shown as the mean ± standard deviation.

### Magnetic Resonance Imaging Parameters

All volumes were acquired with a 3.0-T MR imager (SIGNA PET/MR; GE Healthcare). Functional volumes were acquired using T2*-weighted gradient-echo echo-planar imaging sequences (53 oblique slices, 3.0 mm thickness, 50% gap, repetition time [TR] = 3000 ms, echo time [TE] = 25 ms, flip angle [FA] = 90°, field of view = 192 × 192 mm, in-plane resolution = 64 × 64 pixels, and pixel dimension = 3 × 3 mm). Axial slices were acquired in interleaved order. A high-resolution anatomical T1-weighted image was acquired by three-dimensional fast spoiled gradient-recalled acquisition (TR = 8.464 ms, TE = 3.248 ms, FA = 11°, 256 × 256 matrix, voxel dimensions = 1 × 1 × 1 mm).

### Experimental Setup

Visual stimulus presentation and response collection were conducted with the Presentation software (Neurobehavioral Systems) implemented on a Windows-based desktop computer. Visual stimuli were presented on a liquid crystal display monitor and participants viewed visual stimuli via a mirror attached to the head coil of the MRI scanner. Head motion was minimized by placing comfortable but tight-fitting foam padding around each participant’s head.

### Task Procedure

**Figure 1:**
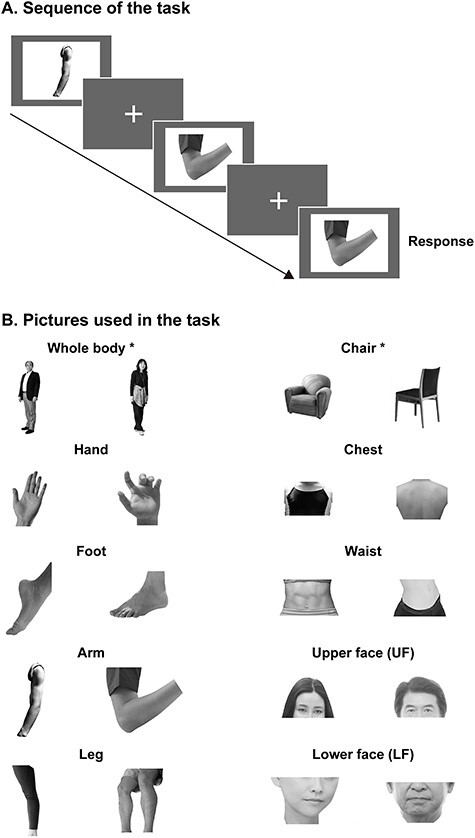
Task procedure. (*A*) The sequence of the task is shown. Participants were asked to press a button when the same picture was presented again. Pictures were presented for 500 ms with a 500-ms interstimulus interval. (*B*) Pictures used in the study are presented. Asterisks indicate pictures used to identify the region of interest.

The task described in Bracci et al. (2015) was modified, as shown in Figure 1, because participants in this study included children and adolescents. Specifically, repetitions were reduced from 8 to 4 runs and the duration of a run was changed from 352 to 318 s. In the task, participants observed greyscale pictures of 10 conditions (whole body, chairs, hands, feet, arms, legs, chests, waists, UFs, and LFs). A total of 440 pictures (44 pictures × 10 conditions = 440 pictures) were prepared. Pictures of each condition, except for the chair condition, included the entire bodies or body parts of 11 men, 11 women, 11 boys, and 11 girls. All pictures were changed to greyscale with a white background color and a matrix size of 800 × 600 pixels using Adobe-Photoshop software (Adobe System Inc.). All participants completed 4 runs; each run included 20 task blocks (10 conditions × 2 repetitions = 20 blocks) and each block lasted for 12 s. The order of the conditions was pseudo-randomized. A fixation-only baseline condition was inserted before the 1st block (27 s), after the 5th, 10th, and 15th block (12 s), and after the 20th block (15 s). In each block, 12 pictures were presented for 500 ms with a 500-ms interstimulus interval. Thus, each run lasted for 318 s (106 volumes per run). The participants were required to complete a 1-back task, for example, pressing a button with the right hand when the same pictures were presented in succession. Target pictures were presented once per block.

### Behavioral Data Analysis

The correct response percent, false-alarm ratio, and response ratio of all stimuli in the 1-back task were calculated for each participant. SPSS software (IBM Corp.) was used to statistically compare the 2 groups with the two-sample *t*-test.

### Functional Magnetic Resonance Imaging Data Analysis

#### Preprocessing

The first 5 volumes of each run were discarded because of unstable magnetization. The remaining 404 volumes (101 volumes × 4 runs = 404 volumes) were analyzed using Statistical Parametric Mapping software (SPM12; Wellcome Department of Imaging Neuroscience; Friston et al. 2002) implemented in MATLAB (MathWorks). After functional image realignment, the high-resolution anatomical images were co-registered to the mean image of the realigned functional images and normalized to a tissue probability map that was already fitted to the Montreal Neurological Institute (MNI) space via a segmentation–normalization procedure. The parameters from this normalization process were then applied to all functional images, which were resampled at a final resolution of 2 × 2 × 2 mm^3^. Its normalized unsmoothed images were utilized for the region of interest (ROI) analysis. The normalized functional images were smoothed using a Gaussian kernel of 8 mm full-width at half-maximum in the *x*-, *y*-, and *z*-axes, which were used for localizing the whole-body person-sensitive region in the LOTC.

#### Statistical Analysis

*Localizing the whole-body sensitive region in the lateral occipitotemporal cortex*. To localize the whole-body sensitive region in the LOTC, a classical mass-univariate analysis was conducted at 2 levels. In the first-level single-subject analysis, a general linear model was fitted to the fMRI data of each participant (Friston et al. 1994; Worsley and Friston 1995). The blood oxygen level-dependent signal was modeled with box-car functions convolved with the canonical hemodynamic response function. Each run included 1 regressor for each of the 10 conditions (whole body, chairs, hands, feet, arms, legs, chests, waists, UFs, and LFs) and 6 regressors of motion-related parameters (3 displacements and 3 rotations obtained by the rigid-body realignment procedure). The time series for each voxel was high-pass filtered at 1/128 Hz. Assuming a first-order autoregressive model, the serial autocorrelation was estimated from a set of pooled active voxels with the restricted maximum likelihood procedure and was used to whiten the data (Friston et al. 2002). Global signal scaling was utilized to remove global confounding, such as a scanner gain change. Parameter estimates of the whole body versus chairs in each participant were compared using linear contrasts.

Next, a second-level group analysis was performed on contrast images from the first-level analyses using the Statistical nonParametric Mapping (SnPM) toolbox (http://warwick.ac.uk/snpm) based on the false-positive ratio indication for cluster inferences using parametric tests (Eklund et al. 2016). Using the one-sample *t*-test, the whole-body sensitive regions were identified in each group and the activation between these 2 groups was compared using the two-sample *t*-test. The mean activation in the child/adolescent group and in the adult group was also depicted to localize the LOTC for the following ROI analysis. The resulting set of voxel values for each contrast constituted the SnPM{T}. The statistical threshold of SnPM{T} was set at *P* < 0.05 corrected for multiple comparisons at the cluster level over the search volume (family-wise error; FWE) with a height (cluster-forming) threshold of *P* < 0.001.

Using a similar procedure to Okamoto et al. (2017), the ratio of participants exhibiting a whole-body person-sensitive activation in the LOTC and the size of its activation were evaluated. An 8-mm radius sphere centered on coordinates reported in a previous study (Bracci et al. 2015), which were transformed from Talairach to MNI coordinates (Lancaster et al. 2007), was assessed for activation. The statistical threshold was set at *P* < 0.01, uncorrected for multiple comparisons. To compare the ratios of participants showing activation and the size of their activation, the χ^2^ test and the two-sample *t*-test were conducted with SPSS software.

*Region of interest analysis*. Using unsmoothed data, the same single-subject analysis was performed as described above for the mass-univariate whole-brain analysis of localizing the whole-body-sensitive region, and the resulting beta image (parameter estimates for each voxel) was used in the following ROI analyses. The mean activation of the child/adolescent group and adult groups (contrast of the whole body vs. a chair) was utilized to rule out the possibility that any between-group differences in activation measures might be confounded by group differences in ROI volumes.

##### Univariate regional average activation analysis

To evaluate the overall activation intensity of the ROIs, mean beta estimates of 8 body parts relative to the baseline were extracted from the ROIs in each hemisphere for each participant. The two-way analysis of variance (ANOVA) on body parts and group was conducted using SPSS software.

*Representational similarity analysis*. A correlation-based RSA was used to evaluate the representational geometry of the neural population code (Haxby et al. 2001; Kriegeskorte and Kievit 2013; Bracci et al. 2015). In each participant, parameter estimates of 8 conditions (i.e., hands, feet, arms, legs, chests, waists, UFs, and LFs) relative to the baseline of each voxel for each run were extracted from the ROIs. Based on the correlation, *r*, among each body part between the parameter estimates of odd and even runs, an 8 condition × 8 condition dissimilarity matrix (1 − *r*) for each ROI was constructed for each participant. The mean dissimilarity matrix was calculated for each group, and multidimensional scaling (MDS) was conducted using the MATLAB function mdscale to visualize similarity structures.

To test whether spatial activation patterns in the LOTC are organized by 3 categories (i.e., action effector body parts, noneffector body parts, and face parts) for both groups, an analysis of similarity (ANOSIM) (Clarke 1993; Legendre and Legendre 1998a, 1998b) was performed for each group using the Fathom Toolbox (Jones 2017) in MATLAB. ANOSIM, which is a hypothesis-driven, nonparametric statistical test widely used in the field of ecology, tests if the similarity between categories is greater than or equal to the similarity within the categories. ANOSIM provides a value of *R*, which is scaled between −1 to +1, where a value close to 1.0 suggests a strong grouping of or high separation between body parts categories. Statistical tests on the *R* values involved a complete permutation test comparing the hypothesized model ([1] hand, arm, leg, and foot, [2] chest and waist, and [3] UF and LF) and 419 alternative models ([1] arm, leg, chest, and waist, [2] UF and LF, [3] foot, hand, and other possible patterns) (i.e., _8_C_4_ × _4_C_2_ = 420 patterns in total). This procedure provides information regarding whether the hypothesized model is highly relevant compared to alternative models. Thus, ANOSIM is more suitable than other approaches, such as hierarchical clustering analysis, for evaluation in this study because a hypothesized clustering model is involved. Interestingly, ANOSIM is commonly used in analyzing multivariate ecological data (Legendre and Legendre 1998a, 1998b). Also, ANOSIM has been used to evaluate the dissimilarity across cell responses in an electrophysiology study (Knaden et al. 2012) and to check subject homogeneity in group analyses in a neuroimaging study (Kherif et al. 2003).

To examine the similarity of spatial activation patterns in the LOTC between the child/adolescent and adult groups, the Mantel test was used that assesses the correlation between 2 dissimilarity matrices (Mantel 1967; Legendre and Legendre 1998a, 1998b; c.f. Handjaras et al. 2016). The test statistic of the Mantel test is the Pearson product-moment correlation coefficient *R*, which falls between −1 and +1. An *R*-value of ±1 suggests a strong positive/negative correlation, respectively. Furthermore, correlation coefficients were calculated between each participant’s dissimilarity matrix and a mean dissimilarity matrix of the participant’s group and between each participant’s dissimilarity matrix and a mean dissimilarity matrix of the other group. Here, the mean dissimilarity matrix of the participant’s group was calculated after excluding his or her dissimilarity matrix. Thus, 4 correlation coefficients were obtained (the correlation between a child/adolescent participant and the child/adolescent group, that between a child/adolescent participant and the adult group, that between the adult participant and the adult group, and that between the adult participant and the child/adolescent group). Then, SPSS software was used to conduct a two-way ANOVA comparing the type of correlation (within-group correlation/between-group correlation) and group (children and adolescents/adults).

Finally, whether the spatial body part organization in the LOTC is associated with several individual features associated with ASD was examined. Because the target age of the DCDQ and MABC-2 is limited to children and adolescents, this correlation analysis was conducted only on the child/adolescent group. To begin the evaluation, the *R* value of the ANOSIM for LOTC ROIs was calculated for each participant and used as a measure for the degree of the spatial body part organization. Then, the correlation analysis based on Spearman’s rho was conducted between *R* values and scores measuring autistic traits (SRS and AQ total scores), sensory characteristics (SP low registration, SP sensory seeking, SP sensory sensitivity, and SP sensation avoiding), motor skills (DCDQ and MABC-2 total scores), age, and the intellectual ability (FSIQ) in each hemisphere. SPSS software was used for this analysis and Bonferroni correction was applied to control the error rate.

*Classification-based multivariate pattern analysis*. The different spatial activation patterns between categories (i.e., action effector and face vs. noneffector body parts) in the LOTC were confirmed using a support vector machine (SVM)-based classification analysis, in addition to the RSA. The Decoding Toolbox (TDT) (Hebart et al. 2015), implemented in SPM12, was utilized. The beta values were used for feature vectors and a linear SVM classification with a leave-one-run-out cross-validation strategy was conducted. As this study utilized an unbalanced design (i.e., the numbers of conditions vary among categories), repeated subsampling (i.e., bootstrapping) was used during the cross-validation iteration based on the template script for unbalanced data (i.e., decoding_template_unbalanced_data.m) provided by the TDT. The number of bootstrap samples was set to 100. To evaluate the significance of the classification performance, the value of the classification accuracy minus chance level was calculated for each category for LOTC ROIs. Whether the decoding accuracies were higher than the levels expected by chance was examined using the one-sample *t*-test in SPSS and comparisons between the 2 groups utilized a two-sample *t*-test. Furthermore, using complete permutation testing, it was further confirmed whether, in each group, the spatial activation patterns in the LOTC can be categorized into the 3 classes: action effector, face, and noneffector. The condition labels belonging to each class (or category) were permuted to obtain an empirical null distribution, which was used to make inferences about the likelihood of the original (hypothesized) labeling. This gave a *P* value of statistical significance for the three-class categorization. In this permutation operation, all 420 (_8_C_4_ × _4_C_2_) possible combinations were considered.

## Results 

### Behavioral Results of the 1-back Task

**Figure 2:**
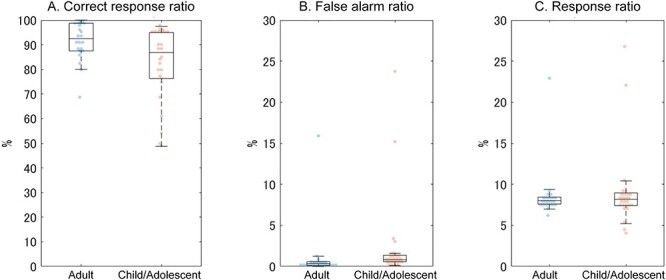
Behavioral performance. The (*A*) correct response ratio for target stimuli, (*B*) false-alarm ratio, and (*C*) response ratio for all stimuli during the 1-back task are shown. Blue: adult group, red: child/adolescent group.

Due to technical problems, behavioral performance could not be measured in 1 out of 4 runs for 2 participants of the child/adolescent group. Thus, the behavioral performance was calculated using the remaining 3 runs in these 2 participants. According to the two-sample *t*-test, the correct response percentage was significantly higher in the adult than the child/adolescent group (*t*_28.68_ = 3.267, *P* = 0.003, Cohen’s *d* = 1.02; Fig. 2). By contrast, there were no group differences in the false-alarm ratio (*t*_46_ = 1.326, *P* = 0.191, Cohen’s *d* = 0.39) or the response ratio for all stimuli (*t*_46_ = 0.512, *P* = 0.611, Cohen’s *d* = 0.15).

### Functional Magnetic Resonance Imaging Results

#### Localizing the Whole-Body-Sensitive Region in the Lateral Occipitotemporal Cortex

**Figure 3:**
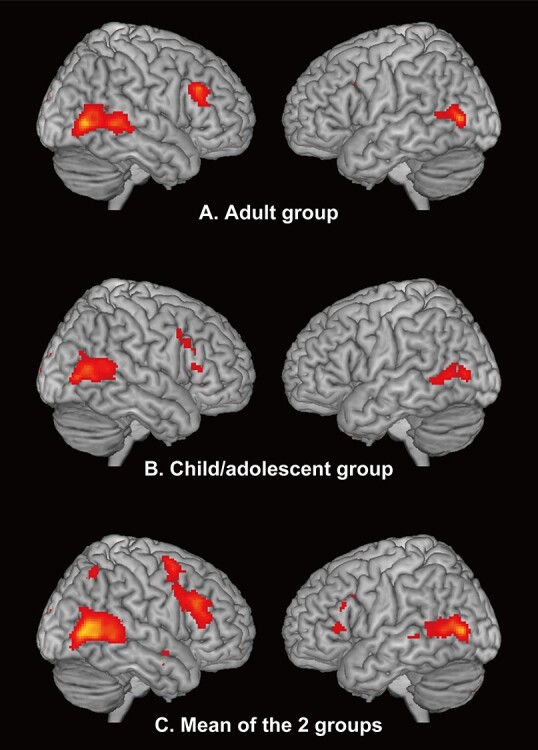
Whole-brain analysis: the whole-body sensitive regions in the child/adolescent and adult groups. Whole-brain person-sensitive regions (the whole body vs. a chair) in the (*A*) adult group, (*B*) child/adolescent group, and the (*C*) mean of the 2 groups superimposed on a T1-weighted magnetic resonance image are shown. The size of the activation was set at a threshold of *P* < 0.05 and corrected for multiple comparisons, with the height threshold set at *P* < 0.001. No brain region revealed a significant group difference.

The contrast between the whole body and a chair in the group analysis revealed bilateral LOTC activation in both groups (Fig. 3*A*,*B*). No brain region showed a group difference. Activation revealed by the mean of the 2 groups was utilized in the following ROI analyses (Fig. 3*C*). Upon examining the whole-body-person-sensitive region in each individual (at the height threshold of *P* < 0.01 uncorrected for multiple comparisons), 26 out of 26 participants of the adult group (100%) and 21 out of 22 participants of the child/adolescent group (95%) exhibited activation in the right hemisphere; there was no group difference (χ_1_^2^ = 1.207, *P* = 0.272, Cramer’s *V* = 0.16). In the left hemisphere, the percentage of participants showing activation was higher in the adult than in the child/adolescent group (adult group: 26 out of 26 participants, 100%; child/adolescent group: 17 out of 22, 77%; χ_1_^2^ = 6.596, *P* = 0.010, Cramer’s *V* = 0.37). Three other height thresholds were used to confirm this result. When using *P* < 0.001, uncorrected, and *P* < 0.05 FWE-corrected for multiple comparisons, the percentage of participants showing activation in the left LOTC was significantly greater in the adult group than in the child/adolescent group (all *P* values <0.05; Supplementary Table 1). There was no group difference for the activation size in each hemisphere (*t*_45_ = 1.175, *P* = 0.246, *d* = 0.35 for the right hemisphere; *t*_41_ = 1.134, *P* = 0.263, *d* = 0.36 for the left hemisphere; Table 2).

**Table 2:** Individual analysis: whole-body sensitive region in the child/adolescent and adult groups

		Peak coordinates			Size (mm^3^)	Ratio and percent	
		*x*	*y*	*z*
L.LOTC	Adult	−50.1 ± 0.4	−73.1 ± 0.3	8.9 ± 0.4	983.1 ± 118.1	26/26	100%
	Child/adolescent	−50.1 ± 0.5	−75.1 ± 0.6	9.2 ± 0.5	774.1 ± 138.9	17/22	77%
R.LOTC	Adult	49.6 ± 0.3	−69.1 ± 0.3	3.3 ± 0.3	954.2 ± 119.7	26/26	100%
	Child/adolescent	49.0 ± 0.5	−70.3 ± 0.4	3.9 ± 0.5	750.1 ± 124.1	21/22	95%

Notes: The LOTC was defined by spheres with an 8-mm radius centered on the peak coordinates of the brain region depicting the whole body versus a chair in Bracci et al. (2015) (height threshold *P* < 0.01). Each value is presented as the mean ± the standard error of the mean. L, left; R, right.

#### Region of Interest Analysis

**Figure 4:**
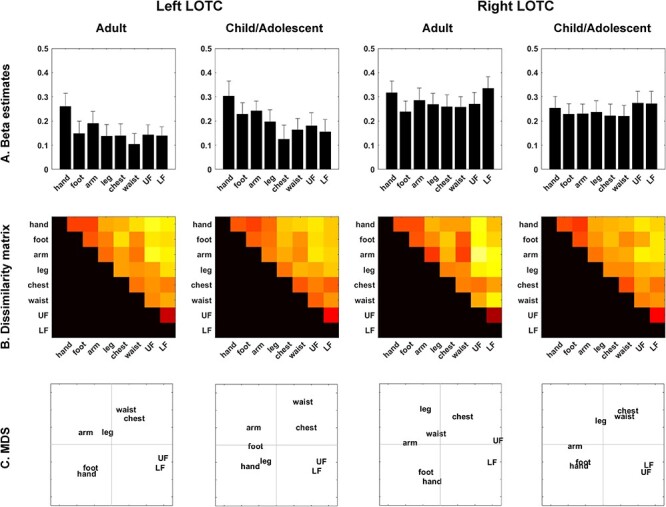
Region of interest analysis for the child/adolescent and adult groups. (*A*) The mean beta estimates of each body part relative to the baseline are shown. (*B*) The dissimilarity matrices (1 − *r*) are presented. (*C*) Multidimensional scaling (MDS) data are depicted. LOTC: lateral occipitotemporal cortex, UF: upper face, LF: lower face.

*Univariate regional average activation analysis*. To evaluate the overall activation intensity in the ROIs, mean beta estimates of 8 body parts (hand, foot, arm, leg, chest, waist, UF, and LF) relative to the baseline were extracted in each ROI (Fig. 4*A*). The two-way ANOVA on body parts and group revealed a significant main effect of body part (right: *F*_7,46_ = 2.492, *P* = 0.017, pη^2^ = 0.051; left: *F*_7,46_ = 7.718, *P* < 0.001, pη^2^ = 0.114). There was no significant main effect of group (right: *F*_7,46_ = 0.626, *P* = 0.735, pη^2^ = 0.013; left: *F*_7,46_ = 0.643, *P* = 0.720, pη^2^ = 0.014) or interaction of group and body part (right: *F*_7,46_ = 0.375, *P* = 0.544, pη^2^ = 0.008; left: *F*_7,46_ = 0.463, *P* = 0.500, pη^2^ = 0.010). A post hoc pair-wise comparison with Bonferroni correction revealed significant differences in the chest versus LF for the right hemisphere and in the hand versus foot, hand versus leg, hand versus chest, hand versus waist, hand versus UF, hand versus LF, and arm versus waist for the left hemisphere (all *P* values <0.05). Thus, the overall activation intensity in the bilateral LOTC was similar between the child/adolescent and adult groups.

**Figure 5:**
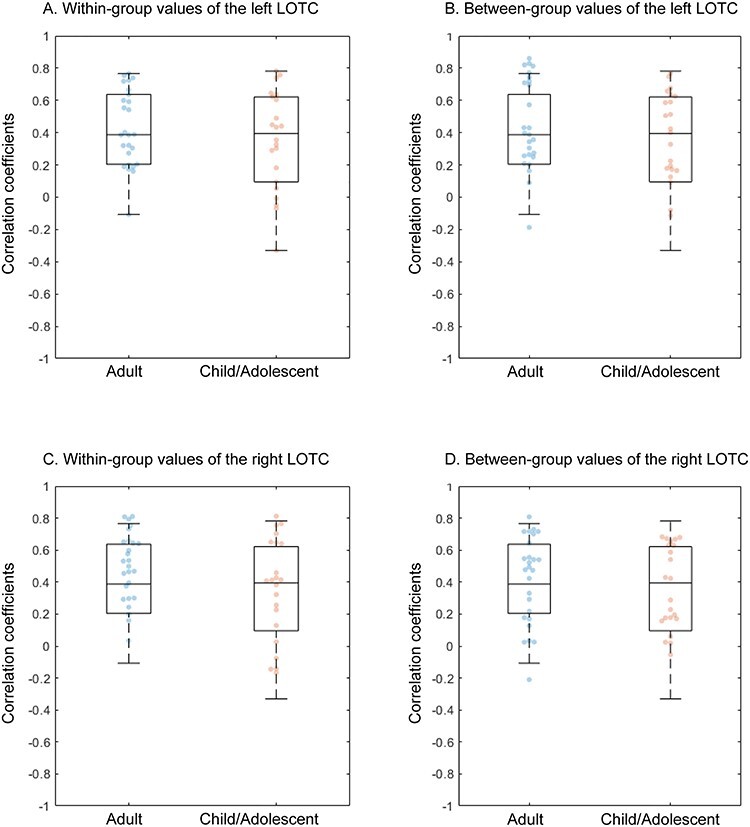
Correlation coefficients between each participant’s dissimilarity matrix and average dissimilarity matrix within and between groups. The (*A*) correlation coefficients of within-group values in the left LOTC, (*B*) between-group values in the left LOTC, (*C*) within-group values in the right LOTC, and (*D*) between-group values in the right LOTC are shown. LOTC, lateral occipitotemporal cortex.

*Representational similarity analysis*. Next, the spatial activation patterns were examined in the LOTCs of both groups. Consistent with a previous study (Bracci et al. 2015), MDS in the adult group showed that action effector body parts (hand, foot, arm, and leg), the face (upper face and lower face), and noneffector body parts (chest and waist) were separately clustered in the bilateral LOTC (Fig. 4*C*). The ANOSIM in the adult group revealed 3 well-characterized clusters in the spatial activation pattern (*R* = 0.738, *P* = 0.012 for the right hemisphere; *R* = 0.863, *P* = 0.002 for the left hemisphere). The child/adolescent group exhibited similar MDS patterns to the adult group (Fig. 4*C*), and the ANOSIM confirmed that the clusters were well-characterized spatial activation patterns (*R* = 0.813, *P* = 0.005 for the right hemisphere; *R* = 0.875, *P* = 0.002 for the left hemisphere). The Mantel test revealed that the dissimilarity matrices of both groups were highly correlated (*R* = 0.795, *P* < 0.001 for the right hemisphere; *R* = 0.898, *P* < 0.001 for the left hemisphere). A two-way ANOVA (correlation type [within-group correlation/between-group correlation] and group [children and adolescents/adults]) on correlation coefficients revealed no significant main effect of correlation type (right: *F*_1,46_ = 2.666, *P* = 0.109, pη^2^ = 0.055, left: *F*_1,46_ = 4.001, *P* = 0.051, pη^2^ = 0.08) or group (right: *F*_1,46_ = 1.624, *P* = 0.209, pη^2^ = 0.034, left: *F*_1,46_ = 0.836, *P* = 0.365, pη^2^ = 0.018) and no interaction of correlation type and group (right: *F*_1,46_ = 3.619, *P* = 0.063, pη^2^ = 0.073, left: *F*_1,46_ = 0.057, *P* = 0.812, pη^2^ = 0.001) (Fig. 5). Head motion, measured by mean frame-wise displacement (FD; Power et al. 2012), was significantly higher in the child/adolescent group (0.19 ± 0.14, mean ± standard deviation) than the adult group (0.10 ± 0.06) (*t*_27.5_ = 2.878, *P* = 0.008, *d* = 0.90). However, the main effect and interaction on the correlation coefficient were not significant when controlling for head motion by an analysis of covariance (all *P* values <0.05). These findings were confirmed by the RSA with an independent LOTC ROI, which was extracted based on a previous study (Bracci et al. 2015; Supplementary Table 2). Therefore, it is unlikely that the results are affected by the procedures to localize ROI.

A univariate analysis of whole body versus chair data also revealed other brain regions involved in the perception of whole body images: the middle frontal gyrus (MFG), lingual gyrus, and inferior parietal lobule (IPL). When conducting the same RSA in these brain regions, the spatial activation pattern was also organized by action effector body parts, noneffector body parts, and face in the bilateral MFG, but not in the lingual gyrus and IPL. Dissimilarity matrices were highly similar between the 2 groups in these regions (Supplementary Information 1).

**Figure 6:**
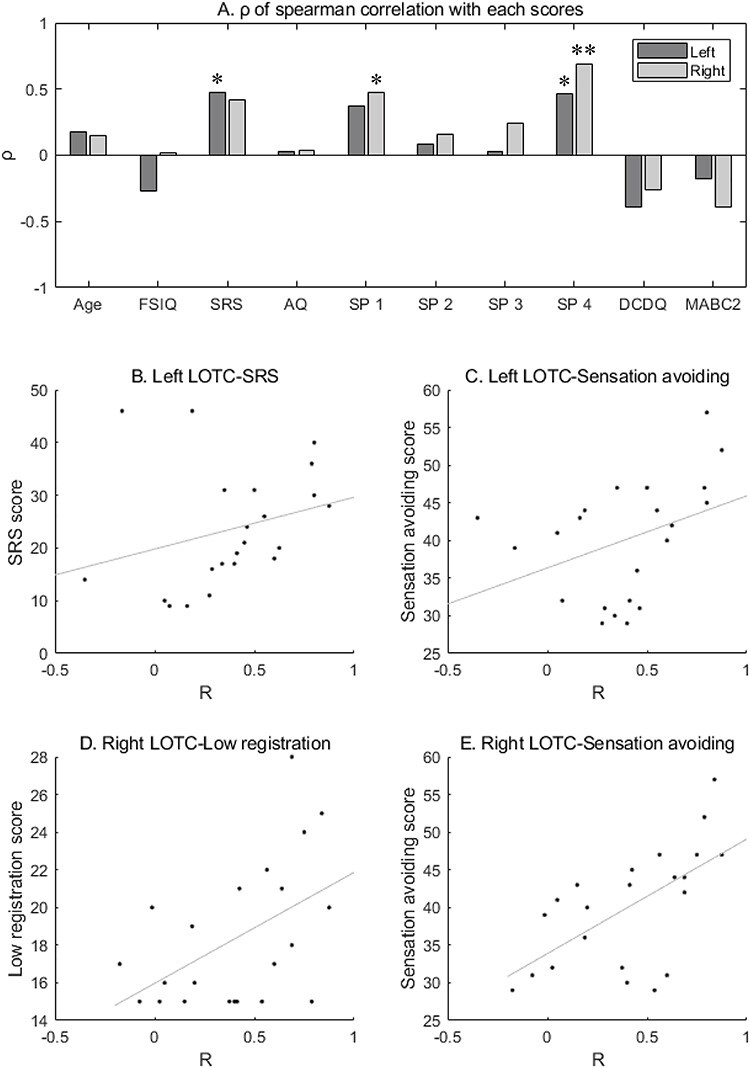
Region-of-interest analysis: correlations between the results of the representational similarity analysis and individual traits in the child/adolescent group. (*A*) The Spearman correlation values (ρ) between the *R* values based on the analysis of similarity and each score are shown. (*B*)–(*E*) Scatter plot of the *R* values and each score are presented. ^*^P < 0.05 uncorrected for multiple comparisons, ^**^P < 0.05/10 with Bonferroni correction, SP1: low registration, SP2: sensory seeking, SP3: sensory sensitivity, SP4: sensation avoiding, FSIQ: full-scale intelligence quotient, SRS: social responsibleness scale, AQ: autism spectrum quotient, DCDQ: developmental coordination disorder questionnaire, and MABC-2: movement assessment battery for children, second edition.

Finally, the associations between the representational geometry in the LOTC and individual traits in the child/adolescent group were examined. The Spearman correlation between ANOSIM *R* values and each score revealed significant correlations for the categories SP low registration (ρ_20_ = 0.476, *P* = 0.025) and SP sensation avoiding (ρ_20_ = 0.691, *P* < 0.001) for the right hemisphere, as well as the SRS (ρ_20_ = 0.470, *P* = 0.027) and SP sensation avoiding (ρ_20_ = 0.462, *P* = 0.030) values for the left hemisphere (Fig. 6). When using Bonferroni correction among scores (0.05/10 scores = 0.005), the ANOSIM *R* values of the right hemisphere were significantly correlated with the scores in the SP sensation avoiding category. The SP sensation avoiding score was not correlated with head motion measured by mean FD or the percent of correct responses in the 1-back task (all *P* value >0.05). When controlling the mean FD by partial correlation analysis, a significant correlation was still observed between the SP sensation avoiding score and *R* values of the right LOTC (*r*_19_ = 0.534, *P* = 0.013).

**Figure 7:**
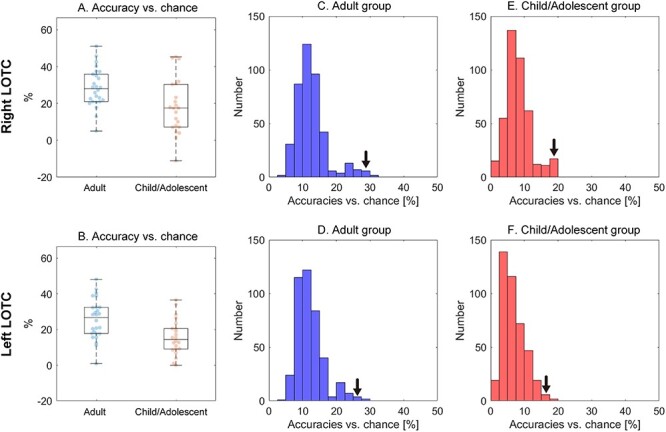
Classification analysis. (*A*) and (*B*) Classification accuracies versus chance (33.3%) in the LOTC are shown. (*C*)–(*F*) The distribution of classification accuracies versus chance calculated based on the 419 possible combinations of the conditions are depicted. Arrows indicate classification accuracies versus chance based on the 3 categories reported in Bracci et al. (2015), that is, action effectors, faces, and noneffector body parts.

*Classification-based multivariate pattern analysis in the LOTC*. As shown in Figure 7*A*,*B*, classification accuracies were significantly higher in the adult group than in the child/adolescent group (right: *t*_46_ = 2.777, *P* = 0.008, *d* = 0.82; left: *t*_46_ = 3.251, *P* = 0.002, *d* = 0.96), although the classification accuracies were above chance level in the adult group (right: *t*_25_ = 13.893, *P* < 0.001, *d* = 2.72; left: *t*_25_ = 11.795, *P* < 0.001, *d* = 2.31) and child/adolescent group (right: *t*_21_ = 5.764, *P* < 0.001, *d* = 1.23; left: *t*_21_ = 7.500, *P* < 0.001, *d* = 1.60). These group differences were reduced in significance after controlling for the mean FD by ANCOVA (right: *P* = 0.046, left: *P* = 0.017), which indicates that lower classification accuracy in child/adolescent can be partially explained by the increased noise contribution such as head motion. However, as shown in Figure 7*C*−*F*, the spatial activation patterns in the LOTC were well-characterized by 3 classes based on action effectors, faces, and noneffector body parts in the child/adolescent group (left: *P* = 0.010; right: *P* = 0.029) as well as in the adult group (left and right: *P* = 0.010).

## Discussion 

In the present study, the percentage of participants showing whole-body-person-sensitive activation in the left LOTC was decreased in the child/adolescent group compared to the adult group. By contrast, the representation of body parts in the bilateral LOTC (i.e., action effectors, noneffectors, and face) was similar between the 2 groups. Furthermore, in the right LOTC, the body part representation was associated with the SP sensation avoiding score but not with age. Thus, these findings provide novel evidence that the body part representation in the LOTC of children and adolescents is similar to that of adults and it contributes to individual differences in sensory processing characteristics.

### Behavioral Performance in the 1-back Task

This study showed that the percentage of correct responses in the 1-back task was lower in the child/adolescent group than in the adult group. As the false-alarm and response ratios for all stimuli were equivalent between these groups, it is unlikely that this decrease in correct responses in the child/adolescent group is due to a failure to perform the button-press task. This finding is consistent with behavioral studies examining developmental changes in body recognition (Weigelt et al. 2014; Bank et al. 2015). Therefore, lower correct responses in the child/adolescent group may reflect that body recognition skills are immature in children and adolescents compared to adults.

### The Lateral Occipitotemporal Cortex

#### Development of Lateral Occipitotemporal Cortex Function

The univariate analysis of the whole-body-person-sensitive region revealed a group difference; an increased percentage of children and adolescents (relative to adults) did not exhibit a whole-body-person-sensitive region in the left LOTC. However, there was no significant difference in the spatial extent of this region, which is inconsistent with previous studies. In the study by Ross et al. (2014), the participants observed videos of humans moving in a socially meaningful manner. Therefore, brain activation may contain information also representing action kinematics and/or meaning rather than simply reflecting body perception. Studies using static pictures reported equivalent (Pelphrey et al. 2009) and larger (Peelen et al. 2009) EBA sizes when face-related activation was subtracted from activation related to nonface body parts. As the activation in the LOTC depends on the type of perceived body parts (Orlov et al. 2010; Bracci et al. 2015), it is reasonable that a distinct task procedure (e.g., picture/movie or action/nonaction) might lead to inconsistent results regarding developmental changes in the spatial extent of body-sensitive activation in the LOTC.

By contrast, the spatial representation of each body part in the LOTC revealed by RSA was similar between the child/adolescent and adult groups. More specifically, the spatial activation patterns of the bilateral LOTC were organized in 3 clusters: (1) action effector body parts (hands, feet, arms, and legs), (2) body parts associated with communication (upper and lower face parts), and (3) noneffector body parts (chests and waists) in both groups; this organization principle was not associated with the age of the participants in the child/adolescent group. Although Ross et al. (2014) performed a multivariate analysis to examine spatial activation patterns in the EBA in adolescents and adults, they only show that their viewing patterns of a body in comparison with a nonbody part were equivalent, but they did not examine the organization for individual body parts. Thus, this study provides novel evidence that body part representation in the LOTC of children and adolescents is organized into 3 clusters, similar to that of adults.

Then, why do the whole-body sensitive and body part representation regions differ in their developmental course? A study of object representation in macaque monkeys suggests that the inferior temporal cortex contains finer columns revealing different functional features like mosaic tiles forming a larger picture of category selectivity (Sato et al. 2013). Thus, it is reasonable that the representational structure of the body in the LOTC appears similar in children, adolescents, and adults. However, neural populations in this region might be more organized such that they are more clustered by body parts during development. In other words, the LOTC in adults involves more neurons responsible for body recognition than in children and adolescents; however, the sensitivity of these neurons to each body part is similar for children, adolescents, and adults. If this is the case, rearranging the massive mosaic structure, making a body-selective region, might lead to improvements in body recognition skills from childhood to adulthood (Weigelt et al. 2014; Bank et al. 2015).

#### Individual Traits and Representational Geometry of Body Parts in the Child/Adolescent Lateral Occipitotemporal Cortex

The spatial organization of body parts in the right LOTC was associated with the SP sensation avoiding score. SP sensation avoiding means attempting to decrease a sensation with higher scores reflecting greater symptoms (Ito et al. 2013) and is frequently observed in individuals with ASD (Dunn 1999; Ito et al. 2013). Furthermore, this sensory atypicality was remarkable in individuals with ASD as compared with individuals with learning difficulties (O'Brien et al. 2009). Thus, the present results suggest that TD children and adolescents who show clear body part representation clustering in the LOTC often attempt to decrease sensation; that is, excessively fine-tuned body part representation in the LOTC might induce sensation avoidance behavior.

### Limitations 

Three limitations of the study should be noted. First, the number of runs in the present study is rather low to conduct a classification analysis. However, a higher number of runs would cause an excessive workload for children and adolescents and can lead to ethical or health problems. Therefore, the approach in the present study is considered valid for interpretation. Second, evidence is provided indicating that children and adolescents have body part representation in the LOTC, and the representation is similar to that of adults. However, as few participants were enrolled, it is difficult to detect slight differences in LOTC body part representation between the 2 groups. Future studies utilizing a larger sample size, perhaps several hundred participants, can address whether the LOTC body part representation is completely identical. Importantly, this approach with various tasks also helps elucidate the controversy of developmental changes via a univariate analysis. Third, the correlation analysis was conducted in TD individuals but not in individuals with ASD; thus, a study examining the association between ASD symptoms and body part representation in the LOTC in individuals with ASD is necessary.

## Conclusions 

The present study demonstrates that body part representation in the LOTC is organized into 3 clusters (i.e., action effector body parts, noneffector body parts, and face parts) in children and adolescents; these clusters are similar to those seen in adults. Furthermore, children and adolescents having clearly distinct body part organization in the LOTC are more likely to attempt to decrease sensation; this contributes to some behavioral traits associated with ASD.

## Notes 

We thank H. Oikawa for acquiring MRI data. *Conflict of Interest*: None declared.

## Funding 

This work was supported by JSPS KAKENHI Grant Numbers JP17K17766, JP20H04272.

## Supplementary Material

Supplementary_tgaa007
