# Supplementary material for: Visual Body Part Representation in the Lateral Occipitotemporal Cortex in Children/Adolescents and Adults
Source: Cereb Cortex Commun. 2020 Apr 13;1(1):tgaa007. doi: 10.1093/texcom/tgaa007 (PMC8152859; doi:10.1093/texcom/tgaa007)
Supplement: Supplementary_tgaa007 [file supplementary_tgaa007.docx]

# Supplementary Table S1. Ratio and percent of participants showing whole-body sensitive activation in the LOTC with various statistical thresholds

|  |  | Adult | | Child and adolescent | | χ^2^ | p | | Cramer's V |
| --- | --- | --- | --- | --- | --- | --- | --- | --- | --- |
| L.LOTC | p < 0.05 | 26/26 | 100% | 19/22 | 86% | 3.782 | 0.052 |  | 0.281 |
|  | p < 0.01 | 26/26 | 100% | 17/22 | 77% | 6.596 | 0.010 | ** | 0.371 |
|  | p < 0.001 | 24/26 | 92% | 14/22 | 64% | 5.939 | 0.015 | * | 0.352 |
|  | p < 0.05 (FWE) | 19/26 | 73% | 6/22 | 27% | 10.018 | 0.002 | ** | 0.457 |
| R.LOTC | p < 0.05 | 26/26 | 100% | 22/22 | 100% | - | - |  | - |
|  | p < 0.01 | 26/26 | 100% | 21/22 | 95% | 1.207 | 0.272 |  | 0.159 |
|  | p < 0.001 | 26/26 | 100% | 18/22 | 82% | 5.157 | 0.023 | * | 0.328 |
|  | p < 0.05 (FWE) | 16/26 | 62% | 7/22 | 32% | 4.218 | 0.040 | * | 0.296 |

* indicates p < 0.05 and ** indicates p < 0.05/4 (Bonferroni correction of 4 statistical thresholds). LOTC: lateral occipitotemporal cortex, L: left, R: right, FWE: family-wise error

# Supplementary Table S2. Representational similarity analysis within the independently defined LOTC

|  | ANOSIM | | | | |  | Mantel test | |
| --- | --- | --- | --- | --- | --- | --- | --- | --- |
|  | Adult | |  | Child and adolescent | |  |  |  |
|  | R | p |  | R | p |  | R | p |
| R.LOTC | 0.925 | 0.005 |  | 0.838 | 0.005 |  | 0.800 | < 0.001 |
| L.LOTC | 0.838 | 0.005 |  | 0.838 | 0.010 |  | 0.920 | < 0.001 |

The bilateral LOTC was defined by spheres with an 8-mm radius centred on the peak coordinates of the brain region depicting the whole body vs. chairs in Bracci et al. (2015), a procedure also used to identify the whole-body person-sensitive region in each individual in the present study. ANOSIM: analysis of similarity, LOTC: lateral occipitotemporal cortex, L: left, R: right

# Supplementary information 1: Representational similarity analysis in the frontal, parietal, and occipital cortices.

The univariate analysis of the whole body vs. a chair revealed the activation of brain regions other than the LOTC, such as the lingual gyrus (x = -6, y = -82, z = 0) overlapped with the primary and secondary visual cortices according to the anatomy toolbox, the right inferior parietal lobule (IPL; x = 42, y = -58, z = 54), the right middle frontal gyrus (MFG; x = 52, y = 26, z = 28), and the left MFG (x = -48, y = 20, z = 28). When conducting the representational similarity analysis for these regions, the analysis of similarity (ANOSIM) revealed that the spatial activation patterns in the bilateral MFG, not in the lingual gyrus and IPL, were organized by (1) action effector body parts, (2) non-effector body parts, and (3) face parts. In all regions, the Mantel test revealed that the spatial representation was similar between the child/adolescent and adult groups.

|  | ANOSIM | | | | |  | Mantel test | |
| --- | --- | --- | --- | --- | --- | --- | --- | --- |
|  | Adult | |  | Child/adolescent | |  |  |  |
|  | R | p |  | R | p |  | R | p |
| Lingual gyrus | 0.125 | 0.314 |  | 0.313 | 0.112 |  | 0.501 | 0.008 |
| R.IPL | 0.425 | 0.050 |  | 0.238 | 0.190 |  | 0.584 | 0.004 |
| L.MFG | 0.775 | 0.002 |  | 0.588 | 0.021 |  | 0.604 | 0.002 |
| R.MFG | 0.813 | 0.005 |  | 0.675 | 0.010 |  | 0.760 | 0.002 |


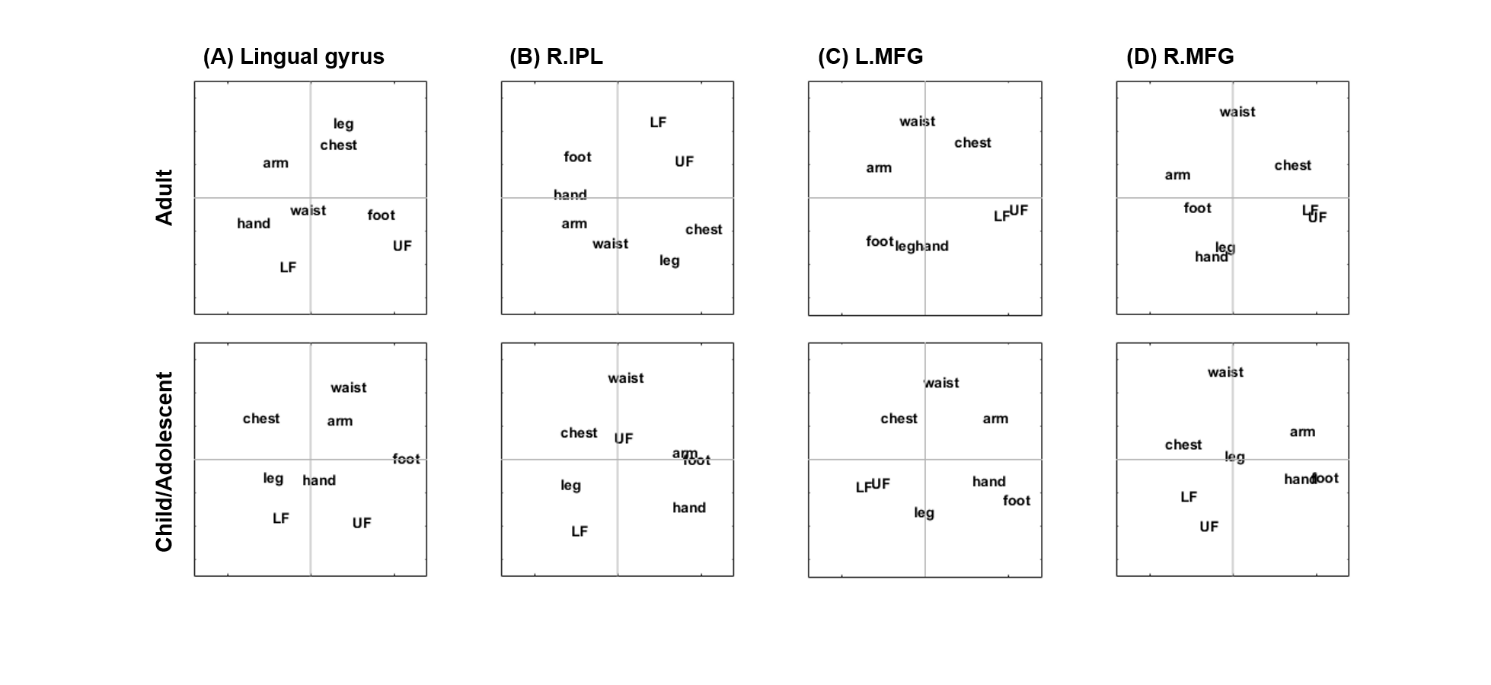


L: left, R: right, UF: upper face, LF: lower face
